# Simultaneous quantification of Bi(III) and U(VI) in environmental water samples with a complicated matrix containing organic compounds

**DOI:** 10.1007/s10661-012-2963-8

**Published:** 2012-10-30

**Authors:** Malgorzata Grabarczyk, Anna Koper

**Affiliations:** Faculty of Chemistry, Maria Curie-Sklodowska University, 20-031 Lublin, Poland

**Keywords:** Bismuth(III), Uranium(VI), Trace analysis, Adsorptive stripping voltammetry, Cupferron, Water samples

## Abstract

Trace amounts of bismuth(III) and uranium(VI) can be simultaneously determined in a single scan by adsorptive cathodic stripping voltammetry in the presence of cupferron as a complexing agent. Optimal conditions were found to be: 0.1 mol L^−1^ acetate buffer (pH 5.3), 5 × 10^−5^ mol L^−1^ cupferron, accumulation potential of −0.25 V, and accumulation time of 30 s. The linear range of Bi(III) and U(VI) was observed over the concentration range from 2 × 10^−9^ to 2 × 10^−7^ mol L^−1^ and from 1 × 10^−8^ to 5 × 10^−7^ mol L^−1^, respectively. The influence of the main components of real water samples such as foreign ions and organic substances (surface active substances, humic substances) was precisely investigated. The method was applied to the simultaneous measurements of bismuth and uranium in natural water samples.

## Introduction

Uranium is a ubiquitous element which occurs naturally in the upper layers of the earth’s crust and in surface and groundwater samples. Generally, it is found in groundwater at a concentration below 15 μg L^−1^ (which is the WHO drinking water guideline) in a hexavalent form (Doming 2001; Mehra et al. 2007; Semião et al. 2010) Most of natural uranium is mined for use in energy production in fission reactors and nuclear research reactors. Other applications involve the use of natural or depleted uranium for armor-piercing shells, ship ballast and counterweights for airplanes or as a negative contrast in electron microscopy. Minor historic applications include tile glazes and glass colors (Merian et al. 2004a, b; Gavrilescu et al. 2009).

Bismuth is a rare metal found in the earth’s crust. It is found in its native form and also in minerals such as bismuthite (bismuth sulfide) and bismite (bismuth oxide) and generally shows a valence of 3+. The main use of bismuth is in pharmaceuticals as an anti-ulcer, antibacterial, and radioterapeutic agent and in low-melting-point alloys which are used as fuses (∼ 4,000 tons annually; Shemirani et al. 2005; Das et al. 2006; Merian et al. 2004a, b). The wide use of bismuth does signify that humans and animals are in fact exposed to or are in contact with this heavy metal (Stoltenberga et al. 2000; Itoch et al. 1999). Bismuth is also used in the preparation and recycling of uranium nuclear fuel and has found application as a carrier for 235U or 233U fuel in nuclear reactors, so the simultaneous presence of bismuth and uranium in polluted environmental samples is likely (Merian et al. 2004a, b). Taking this into consideration, procedures for simultaneous determination of these elements are needed.

There are a lot of methods for uranium and bismuth determination. These include inductively coupled plasma–optical emission spectrometry (Sun and Wu 2011; Chandrasekaran et al. 2011); inductively coupled plasma–mass spectrometry (Takata et al. 2011; Krishna and Arunachalam 2004); atomic absorption spectrometry (Zhang and Adeloju 2008; Kumar et al. 2001); and numerous stripping procedures such as potentiometric stripping analysis (Gadhari et al. 2010; Wang et al. 1984), anodic stripping voltammetry (Huang 2004; Pournaghi-Azar et al. 2010) and, most of all, adsorptive stripping voltammetry (AdSV) (Hajian and Shams 2003; Gholivand and Romiani 2006a, b; Khaloo et al. 2007; Shams 2001; Babaei et al. 2006; Novotný et al. 2003; Lin et al. 2005; Kefala et al. 2006; Piech et al. 2007; Korolczuk et al. 2007; Kadi and El-Shahawi 2009; Abbasi et al. 2008). Such a keen demand for adsorptive stripping procedures lies in the capabilities they offer, such as low cost and portable instrumentation, a low detection limit and the possibility of the simultaneous determination of a few elements. In the literature data, a lot of procedures for the simultaneous determination of uranium(VI) with other elements such as zinc, chromium, molybdenum, vanadium, antimony and cadmium (Wang et al. 1997a, b; Sander 1999; Cha et al. 2000; Ahmadi and Bakhshandeh-Saraskanrood 2010) have been described. In the case of bismuth, only procedures for simultaneous determination with copper and lead have been described (Hajian and Shams 2003; Gholivand and Romiani 2006a, b; Khaloo et al. 2007; Babaei et al. 2006). There are no AdSV methods for the simultaneous quantification of uranium and bismuth, whereas trace analysis of these elements is important for monitoring their concentrations in the environment. It stems from the fact that its simultaneous presence in polluted environmental samples is quite possible, as mentioned above.

Considering the great relevance of selective analytical methods for the analysis of environmental samples, this work aims to show the development of a procedure based on adsorptive cathodic stripping voltammetry for the simultaneous determination of bismuth and uranium in water samples. Measurement relies on the complexation of Bi(III) and U(VI) with a cupferron and adsorptive preconcentration of the complexes on the surface mercury working electrode. Following this accumulation step, a cathodic voltammetric scan is applied to reduce the complexes and the reduction currents are related to the concentrations of these elements.

Because the procedure is destined for the analysis of real water samples, the matrix of such samples was taken into account. The main components of real water samples which can interfere during adsorptive voltammetric measurement are foreign ions and organic substances, particularly surface-active substances such as typical industrial pollutants (Wang 1985). Interferences from foreign ions result from the possibility of the formation of their complexes with cupferron, and those complexes can be adsorbed on the working electrode and then reduced, causing enhancement or a decrease of the voltammetric current. In the case of surface-active substances, they tend to adsorb on the mercury electrode, thus inhibiting the deposition step and/or stripping processes, causing a decrease or total decay of the analytical signal (Wang 1985; Hoyer and Jensen 2003). In the proposed procedure, the influence of these interferents was precisely studied and, as the need arose, minimalized.

## Experimental section

### Reagents

The U(VI) solutions for testing and calibration were prepared every day from 1 × 10^−2^ mol L^−1^ uranium stock solution prepared by dissolving (CH_3_COO)_2_UO_2_⋅2H_2_O in 0.1 mol L^−1^ HNO_3_. A stock standard solution of 1 g L^−1^ of Bi(III) and cupferron (*N*-nitrosophenylhydroxylamine ammonium salt) were obtained from Merck (Darmstadt, Germany). Acetate buffer (1 mol L^−1^) was prepared from Suprapur CH_3_COOH and NaOH obtained from Merck. Triton X-100, sodium dodecyl sulfate (SDS) and cetyltrimethylammonium bromide (CTAB) used as nonionic, anionic and cationic surface-active substances were purchased from Fluka (Buchs, Switzerland). River fulvic acid (FA) was a standard sample obtained from the Suwannee River and purchased from the International Humic Substances Society. Humic acid sodium salt was obtained from Aldrich. For the evaluation of the precision and accuracy of the measurement, the standard material “SPS-SW1 Batch no. 116—Reference Material Surface Water Level 1” from Spectrapure Standards AS (Oslo, Norway) was used. Amberlite XAD-16 was obtained from Sigma, washed four times in water and dried up at a temperature of 50 °C. All solutions were made using triply distilled water.

### Instrumentation

All voltammetric measurements were carried out with a μAutolab analyser (Utrecht, the Netherlands). The three-electrode system was completed using a hanging mercury drop electrode made by MTM-ANKO (Cracow, Poland) as a working electrode, platinum wire as an auxiliary electrode and an Ag/AgCl (filled with NaCl) electrode as a reference electrode. The Hg drop area was 1.5 mm^2^. The solutions were deoxygenated with high-purity nitrogen for 5 min prior to each experiment and kept under nitrogen atmosphere during the measurements. All experiments were carried out at room temperature.

### Procedure

Standard voltammetric measurement was performed using the following means. To the analysed sample, e.g. a real water sample or a synthetic sample (containing suitable concentrations of Bi(III) and U(VI) diluted in triply distilled water), 1 mL of 1 mol L^−1^ acetate buffer (pH 5.3), 50 μL of 1 × 10^−2^ mol L^−1^ cupferron and an adequate volume of triply distilled water were added so that the final volume of the solution was 10 mL. After deoxygenation with nitrogen for 5 min, the standard measuring procedure was performed using differential pulse adsorptive cathodic stripping voltammetry. A mercury drop was formed and the accumulation of the Bi(III)–cupferron and U(VI)–cupferron complexes was carried out from the stirred solution at −0.25 V for 30 s. At the end of the accumulation time, the stirrer was switched off; after the equilibration time of 5 s, differential pulse voltammogram was recorded whilst the potential was scanned from −0.1 to −0.5 V. The scan rate and pulse height were 20 mV s^−1^ and −50 mV, respectively.

## Results and discussion

### Optimization of analytical parameters

A previous study has shown that U(VI) and Bi(III) form electrochemically active stable complexes with cupferron, which makes voltammetric determination of these elements with a low detection limit possible (Kefala et al. 2006; Korolczuk et al. 2007; Grabarczyk and Koper 2011a, b; Koper and Grabarczyk 2011). The proposed procedure concentrated on the selection of optimum conditions for the simultaneous determination of uranium and bismuth in one voltammetric scan. To achieve optimum performance, we studied the influence of various experimental parameters (e.g. cupferron concentration, pH, accumulation potential, accumulation time) on the voltammetric curve in order to obtain the best shape and separation of uranium and bismuth peaks recorded on the voltammetric curve.

#### Cupferron concentration

**Fig. 1:**
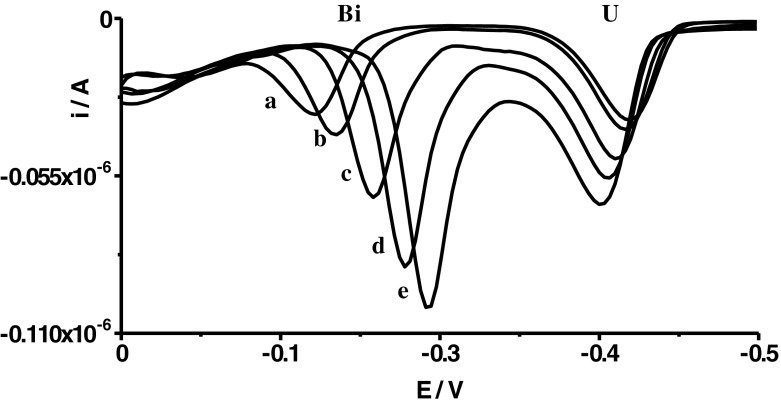
Voltammograms recorded for different concentrations of cupferron: 5 × 10^−6^ mol L^−1^ (*a*); 1 × 10^−5^ mol L^−1^ (*b*); 5 × 10^−5^ mol L^−1^ (*c*); 2 × 10^−4^ mol L^−1^ (*d*); 5 × 10^−4^ mol L^−1^ (*e*). The concentration of Bi(III) was 4 × 10^−8^ mol L^−1^ and that of U(VI) was 1 × 10^−7^ mol L^−1^

**Fig. 2:**
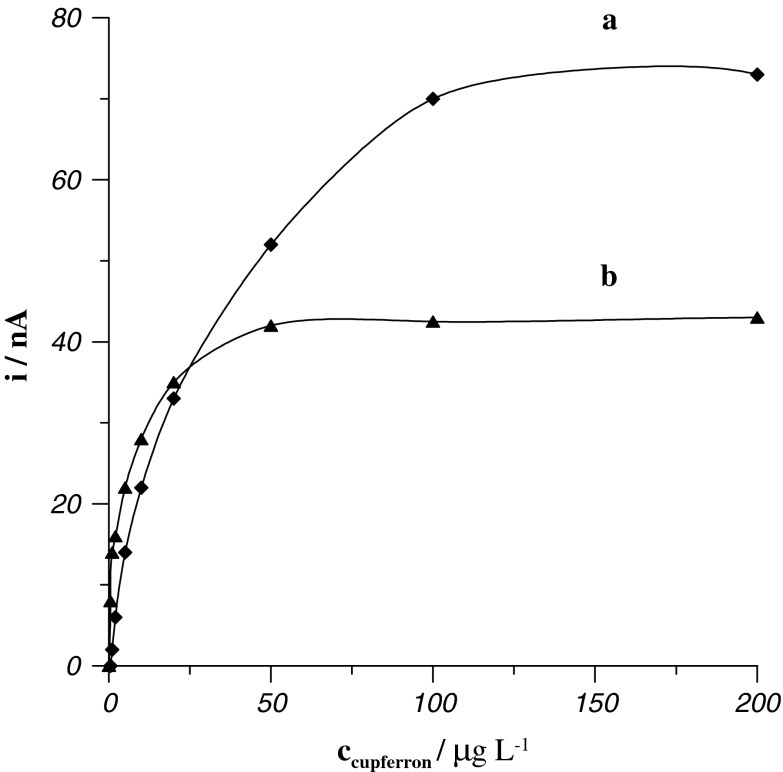
Influence of the concentration of cupferrron on peak currents of 4 × 10^−8^ mol L^−1^ Bi(III) (*a*) and 1 × 10^−7^ mol L^−1^ U(VI) (*b*)

Preliminary experiments indicated that the concentration of cupferron not only influenced the uranium and bismuth peak currents but, in a large part, influenced the potentials of peaks and, consequently, separations between peaks. Exemplary voltammograms recorded for different concentrations of cupferron were presented in Fig. 1. As can be seen, with increasing cupferron concentration, the potential of the bismuth peak moves up in the direction of more negative potentials whereas the potential of the uranium peak moves up in the direction of more positive potentials. In this way, the growth of cupferron concentration enhances the peak currents (Fig. 2), but equally worsens the separation of the uranium and bismuth peaks. As a compromise between sensitivity and separation of peaks, cupferron concentration of 5 × 10^−5^ mol L^−1^ was chosen for further experiments; at this concentration, *E*_p Bi_ ≅ −0.2 mV and *E*_p U_ ≅ −0.4 mV.

#### pH value

**Fig. 3:**
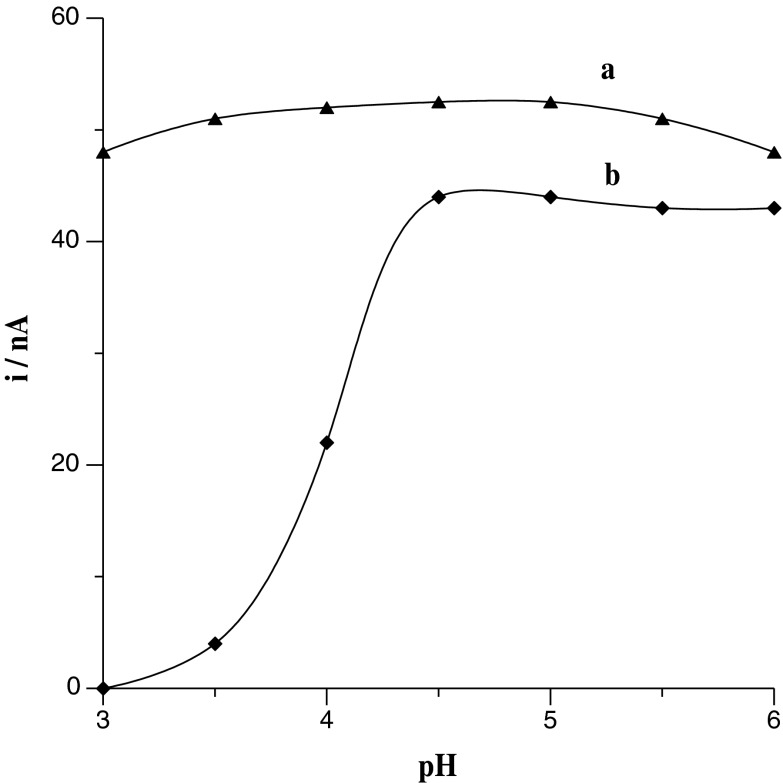
Influence of pH on Bi(III) (*a*) and U(VI) (*b*) peak currents. The concentration of Bi(III) was 4 × 10^−8^ mol L^−1^ and that of U(VI) was 1 × 10^−7^ mol L^−1^

As a supporting electrolyte, an acetate buffer was chosen on the basis of literature data (Korolczuk et al. 2007; Grabarczyk and Koper 2011a, b; Koper and Grabarczyk 2011) as the most suitable both for U(VI)–cupferron and Bi(III)–cupferron complex formation and accumulation on the working electrode. The pH of the supporting electrolyte was changed from 3 to 6, and its influence on uranium and bismuth peak currents is presented in Fig. 3. It was observed that the pH slightly influences the separation of the analysed peaks and that with more acid solution the separation of peaks insignificantly gets worse. Summarizing these data, pH equal to 5.3 ± 0.1 was suggested as the most optimal.

#### Accumulation potential and time

The effect of the accumulation potential on the peak currents of uranium and bismuth was examined over the range from 0.25 to −0.65 V. The measurements were performed for a solution containing 1 × 10^−7^ mol L^−1^ U(VI), 4 × 10^−8^ mol L^−1^ Bi(III), 0.1 mol L^−1^ acetate buffer (pH 5.3) and 5 × 10^−5^ mol L^−1^ cupferrron. It was observed that in the whole studied range of the accumulation potential, the potentials of the uranium and bismuth peaks remain almost the same. In other words, the accumulation potentials do not influence the separation of the examined peaks. The accumulation potential also did not influence peak currents in the range from 0.15 to −0.65 V; only at the potential of 0.25 V was a decrease of peak currents observed. A decrease of peak currents at a more positive potential can result from the partial oxidation of mercury in the presence of the complexing agent. An optimum accumulation potential of −0.25 V was chosen in the proposed procedure.

Accumulation time is one of the important parameters that have a pronounced effect on sensitivity in adsorptive voltammetric stripping procedures. Accumulation time was examined in the 0- to 180-s range at an accumulation potential equal to −0.25 V, whilst other standard measuring conditions remained constant, as described above. The value of the voltammetric peak currents increased almost linearly with accumulation time to 120 and 30 s for uranium and bismuth, respectively.

#### Linear ranges and detection limits

Linear calibration graphs were in the concentration ranges of 1 × 10^−8^–5 × 10^−7^ mol L^−1^ and 2 × 10^−9^–2 × 10^−7^ mol L^−1^ for U(VI) and Bi(III), respectively. The slope of the calibration graph and the relative standard deviation (RSD) of the slope were, respectively, 0.42 nA nmol L^−1^ and 0.7 % for U(VI) and 1.25 nA nmol L^−1^ and 0.6 % for Bi(III). The intercept and the RSD of the intercept were, respectively, 1.42 nA and 1.2 % for U(VI) and 2.56 nA and 0.8 % for Bi(III). The linear correlation coefficients were *r* = 0.9987 and *r* = 0.9989 for U(VI) and Bi(III), respectively. The detection limits estimated from 3 times the standard deviation of low U(VI) and Bi(III) concentrations and accumulation time 30 s were about 3.0 × 10^−9^ and 7.8 × 10^−10^ mol L^−1^, respectively. The RSD from six determinations at a concentration of 5 × 10^−8^ mol L^−1^ of U(VI) was 3.3 % and at 1 × 10^−8^ mol L^−1^ of Bi(III) was 3.5 %. All the measurements were performed under the selected conditions: 0.1 mol L^−1^ acetate buffer (pH 5.3), 5 × 10^−5^ mol L^−1^ cupferron, deposition potential of −0.25 V and deposition time of 30 s.

### Interference

In order to study the influence of the main components of real water samples such as foreign ions and organic substances, which can disturb sensitive and selective determination of Bi(III) and U(VI), a series of measurements as described below was performed.

#### Influence of foreign ions

The effect of potentially interfering ions, which can be distributed in natural samples, was studied using fixed concentrations of 1 × 10^−7^ mol L^−1^ U(VI) and 4 × 10^−8^ mol L^−1^ Bi(III) and different amounts of foreign ions under standard condition. The results are presented as a tolerable limit of foreign ions which was defined as the amount of ions that produced an error not exceeding 5 % in the peak current of the determined elements. The tolerance levels of foreign ions in the determination of Bi(III) were 100 μmol L^−1^ of As(III), As(V), Ca(II), Cd(II), Cr(III), K(I), Mg(II), Mn(II), Na(I) and Se(IV); 10 μmol L^−1^ of Co(II), Fe(III), Ni(II), Se(IV), W(VI) and Zn(II); 1 μmol L^−1^ of Al(III), Cr(VI), Cu(II), Mo(VI), Pb(II), Sb(III) and V(V); and 0.1 μmol L^−1^ of Pb(II). The addition of 1 μmol L^−1^ of Pb(II) causes about a 40 % decrease of the bismuth peak. The tolerance levels of foreign ions in the determination of Bi(III) were 100 μmol L^−1^ of As(III), As(V), Ca(II), Cd(II), Co(II), Cr(III), Cu(II), Fe(III), K(I), Mg(II), Mn(II), Na(I), Ni(II), Se(IV), Se(VI) and Zn(II); 10 μmol L^−1^ of Mo(VI) and W(VI); 1 μmol L^−1^ of Al(III), Cr(VI), Sb(III) and V(V); and 0.1 μmol L^−1^ of Pb(II).

#### Influence of organic substances

Because the aim of the proposed procedure was the simultaneous determination of bismuth and uranium in water samples with special respect to environmental samples, investigation of the influence of organic matter was required. The most important components of organic matter present in natural water samples are surface-active substances such as typical industrial pollutants and humic substances such as the major components of natural organic matter.

The interference of these substances was precisely investigated in previously published papers describing procedures for a separate determination of Bi(III) and U(VI) (Wang et al. 1984; Wang et al. 1997a, b). In order to reduce these interferences, addition of the Amberlite XAD-7 resin (Grabarczyk and Koper 2011a, b; Koper and Grabarczyk 2011) or pulsed potential accumulation (Grabarczyk and Koper 2011a, b) was proposed. In this work, the addition of Amberlite XAD-16 resin was proposed for the elimination of the interference of organic substances. In such measurements, a two-step course was proposed. In the first step, the analysed sample was mixed within 5 min with resin in the presence of an acetate buffer (0.5 g of resin on 10 mL of solution); during this time, the organic substances were removed by adsorption on the resin. In the second step, after sedimentation of resin, an appropriate volume of the sample solution was transferred to the voltammetric cell and standard measurement was performed as described in “Procedure”. The measurements were performed for synthetic solutions containing fixed concentrations of 1 × 10^−7^ mol L^−1^ U(VI) and 4 × 10^−8^ mol L^−1^ Bi(III) and different amounts of Triton X-100, SDS and CTAB as representative nonionic, anionic and cationic surfactants, respectively. It turned out that in the presence of 10 mg L^−1^ of nonionic and cationic surfactants and 5 mg L^−1^ of anionic surfactant, the height and shape of the analysed peaks did not change compared to their signal obtained in the absence of these interferents.

**Fig. 4:**
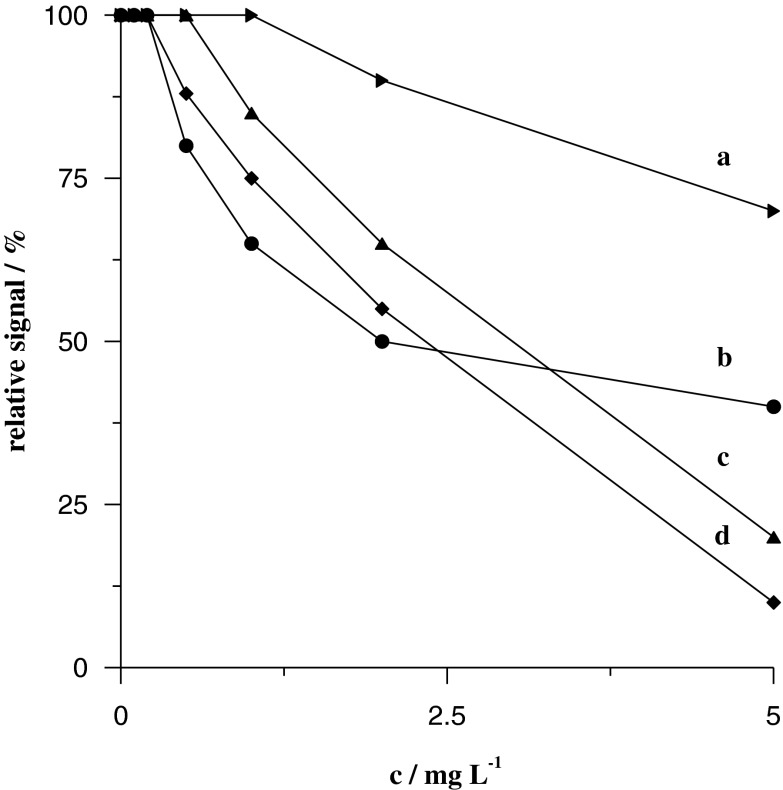
Influence of FA (*a*, *b*) and HA (*c*, *d*) concentrations on peak currents of 1 × 10^−7^ mol L^−1^ U(VI) (*a*, *c*) and 4 × 10^−8^ mol L^−1^ Bi(III) (*b*, *d*)

Humic substances are significant components of natural organic matter inherent in water samples (Potin-Gautier et al. 1995). Thus, the influence of commercially available organic matter, e.g. humic acids (HA) and FAs, was tested in the proposed procedure. The experiments were performed for constant concentrations of U(VI) and Bi(III) and different concentrations of HA and FA in the presence of the XAD-16 resin. The measurements were carried out in the same manner as for surface-active substances. The results presented in Fig. 4 show that humic substances are interfering agents and that the addition of 2.5 mg L^−1^ humic substances led to a decrease of the analysed signals to about 85–50 % of their original values. Thus, it could be concluded that analysis of environmental samples rich in natural organic matter can be performed, but the decrease of the analytical signal and, consequently, the increase of the detection limit must be taken into account.

### Application to real samples

**Fig. 5:**
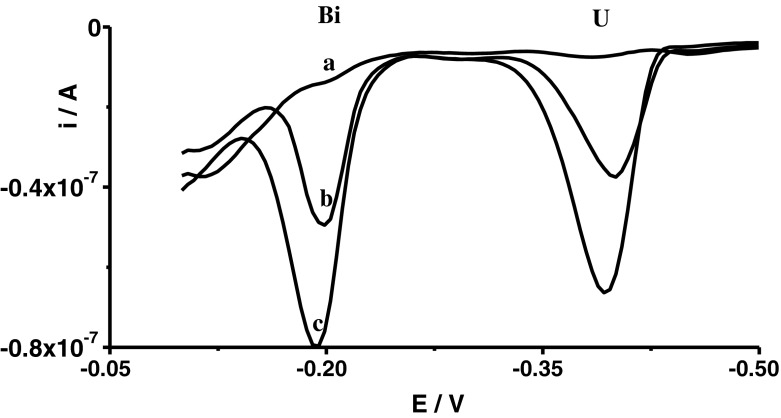
Differential pulse voltammograms obtained in the course of Cr(VI) determination in certified reference material SPS-SW1: SPS-SW1 diluted ten times (*a*); as (*a*) + 2.5 × 10^−8^ mol L^−1^ Bi(III) + 7.5 × 10^−8^ mol L^−1^ U(VI) (*b*); and as (*b*) + 5 × 10^−8^ mol L^−1^ Bi(III) + 1.5 × 10^−7^ mol L^−1^ U(VI) (*c*)

In order to demonstrate the applicability and reliability of the presented procedure for real water samples, tap water and certified reference material of environmental water were chosen. The voltammograms recorded for tap water samples did not exhibit any signal of U(VI) and Bi(III), so spiked experiments were performed. Because of the lack of an environmental water certified reference material containing suitable concentrations of U(VI) and Bi(III), recovery studies were carried out from a certified reference material (SPS-SW1) for the measurement of elements in surface waters. This material contains a lot of different elements, among the other U(VI), but its concentration is below the analytical parameters of the proposed procedure. Tap water and the certified reference material did not undergo any pretreatment and were analysed directly in the form in which they had been purchased after four times and ten times dilutions, respectively. The measurements were performed using the method of standard addition and exploiting previous mixture of the analysed sample with the Amberlite XAD-16 resin, as described in “Influence of organic substances”. The obtained results are presented in Table 1. Figure 5 shows the typical voltammograms obtained during the quantification of U(VI) and Bi(III) in certified reference material SPS-SW1.

**Table. 1:** Results of U(VI) and Bi(III) determination in spiked natural water samples

Sample	Added (nmol L^−1^)		Found (nmol L^−1^)	
	U(VI)	Bi(III)	U(VI)	Bi(III)
Tap water	–	–	ND	ND
	7.5	2.5	7.0 (3.5)	2.7 (3.4)
	15.0	5.0	14.5 (3.7)	4.7 (4.2)
CRM surface waters SPS-SW1	–	–	ND	ND
	7.5	2.5	7.9 (5.6)	2.3 (5.1)
	15.0	5.0	14.2 (5.3)	5.2 (5.3)

Values in parentheses are the relative standard deviations in per cent (*n* = 5)

*ND* not detected

## Conclusion

The present study demonstrates that adsorptive stripping voltammetry with cupferron as a complexing agent is an excellent method of the simultaneous determination of trace amounts of uranium and bismuth. In conclusion, the above method offers a practical potential for water sample analysis, especially with the advantages of high sensitivity, simplicity, speed and low cost. The determination of metal traces in environmental water using adsorptive stripping techniques is normally difficult when natural or anthropogenic organic compounds are present in the sample. This investigation has shown that interference from organic substances can be avoided if preliminary mixing (within 5 min) of the analysed sample with Amberlite XAD-16 resin is employed. For this case, no sample pretreatment in the form of digestion needs to be done. The satisfactory results of the analysis of real samples imply a promising application of the recommended procedure for the simultaneous determination of uranium and bismuth in different kinds of environmental water samples.

## References

[CR1] Abbasi S, Sohrabi A, Naghipour A, Gholivand MB, Ahmadi F (2008). Determination of ultra trace amounts of uranium(VI) by adsorptive stripping voltammetry using L-3-(3,4dihydroxy phenyl) alanine as a selective complexing agent. Analytical Letters.

[CR2] Ahmadi F, Bakhshandeh-Saraskanrood F (2010). Simultaneous determination of ultra trace of uranium and cadmium by adsorptive cathodic stripping voltammetry using H-Point standard addition method. Electroanalysis.

[CR3] Babaei A, Shams E, Samadzadeh A (2006). Simultaneous determination of copper, bismuth and lead by adsorptive stripping voltammetry in the presence of thymolphthalexone. Analytical Scientific.

[CR4] Cha K-W, Park C-I, Park S-H (2000). Simultaneous determination of trace uranium(VI) and zinc(II) by adsorptive cathodic stripping voltammetry with aluminon ligand. Talanta.

[CR5] Chandrasekaran K, Karunasagar D, Arunachalam J (2011). Dispersive liquid–liquid micro extraction of uranium(VI) from groundwater and seawater samples and determination by inductively coupled plasma–optical emission spectrometry and flow injection–inductively coupled plasma mass spectrometry. Analytical Methods.

[CR6] Das AK, Chakraborty R, Cervera ML, de la Guardia M (2006). Analytical techniques for the determination of bismuth in solid environmental samples. Trends in Analytical Chemistry.

[CR7] Doming JL (2001). Reproductive and developmental toxicity of natural and depleted uranium: a review. Reproductive Toxicology.

[CR8] Gadhari NS, Sanghavi BJ, Karna SP, Srivastava AK (2010). Potentiometric stripping analysis of bismuth based on carbon paste electrode modified with cryptand [2.2.1] and multiwalled carbon nanotubes. Electrochimica Acta.

[CR9] Gavrilescu M, Pavel LV, Cretescu I (2009). Characterization and remediation of soils contaminated with uranium. Journal of Hazardous Materials.

[CR10] Gholivand MB, Romiani AA (2006). Application of adsorptive stripping voltammetry to the simultaneous determination of bismuth and copper in the presence of nuclear fast red. Analytica Chimica Acta.

[CR11] Gholivand MB, Romiani AA (2006). Highly sensitive and selective measurement of bismuth in seawater and drug with 1,2-phenylenedioxydiacetic acid by cathodic adsorptive stripping voltammetry. Electroanalysis.

[CR12] Grabarczyk M, Koper A (2011). Adsorptive stripping voltammetry of uranium: elimination of interferences from surface active substances and application to the determination in natural water samples. Analytical Methods.

[CR13] Grabarczyk M, Koper A (2011). How to determine uranium faster and cheaper by adsorptive stripping voltammetry in water samples containing surface active compounds. Electroanalysis.

[CR14] Hajian R, Shams E (2003). Application of adsorptive stripping voltammetry to the determination of bismuth and copper in the presence of morin. Analytica Chimica Acta.

[CR15] Hoyer B, Jensen N (2003). Suppression of surfactant interferences in anodic stripping voltammetry by sodium dodecyl sulfate. Electrochemistry Communication.

[CR16] Huang W (2004). Voltammetric determination of bismuth in water and nickel metal samples with a sodium montmorillonite (SWy-2) modified carbon paste electrode. Microchimica Acta.

[CR17] Itoch S, Kaneco S, Ohta K, Mizuno T (1999). Determination of bismuth in environmental samples with Mg ± W cell ± electrothermal atomic absorption spectrometry. Analytica Chimica Acta.

[CR18] Kadi KW, El-Shahawi MS (2009). Differential pulse cathodic stripping voltammetric determination of uranium with arsenazo-III at the hanging mercury dropping electrode. Radiochimica Acta.

[CR19] Kefala G, Economou A, Voulgaropoulos A (2006). Adsorptive stripping voltammetric determination of trace uranium with a bismuth-film electrode based on the U(VI)–U(V) reduction step of the uranium–cupferron complex. Electroanalysis.

[CR20] Khaloo SS, Ensafi AA, Khayamian T (2007). Determination of bismuth and copper using adsorptive stripping voltammetry couple with continuous wavelet transform. Talanta.

[CR21] Koper A, Grabarczyk M (2011). Electrochemical determination of bismuth using a Bi(III)–cupferron complexation system and elimination of interferences connected with the presence of organic substances in natural samples. Journal of Electroanalytical Chemistry.

[CR22] Korolczuk M, Tyszczuk K, Grabarczyk M (2007). Determination of uranium by adsorptive stripping voltammetry at a lead film electrode. Talanta.

[CR23] Krishna MVB, Arunachalam J (2004). Ultrasound-assisted extraction procedure for the fast estimation of major, minor and trace elements in lichen and mussel samples by ICP-MS and ICP-AES. Analytica Chimica Acta.

[CR24] Kumar M, Rathore DPS, Singh AK (2001). Pyrogallol immobilized Amberlite XAD-2: a newly designed collector for enrichment of metal ions prior to their determination by flame atomic absorption spectrometry. Microchimica Acta.

[CR25] Lin L, Thongngamdee S, Wang J, Lin Y, Sadik OA, Ly SY (2005). Adsorptive stripping voltammetric measurements of trace uranium at the bismuth film electrode. Anaytica Chimica Acta.

[CR26] Mehra R, Singh S, Singh K (2007). Uranium studies in water samples belonging to Malwa region of Punjab, using track etching technique. Radiation Measurements.

[CR27] Merian E, Anke M, Ihnat M, Stoeppler M (2004). Elements and their compounds in the environment.

[CR28] Merian E, Anke M, Ihnat M, Stoeppler M (2004). Elements and their compounds in the environment.

[CR29] Novotný L, Navrátil T, Sander S, Bašová P (2003). Electrocapillary activity and adsorptive accumulation of U(VI)–cupferron and U(VI)–chloranilic acid complexes on mercury electrode. Electroanalysis.

[CR30] Piech R, Baś B, Kubiak WW (2007). The cyclic renewable mercury film silver based electrode for determination of uranium(VI) traces using adsorptive stripping voltammetry. Electroanalysis.

[CR31] Potin-Gautier M, Séby F, Astruc M (1995). Interference of humic substances on the speciation analysis of inorganic selenium in waters and soils by DPCSV. Fresenius’ Journal of Analytical Chemistry.

[CR32] Pournaghi-Azar MH, Dastangoo H, Bajeh RFB (2010). Anodic stripping voltammetric determination of uranium at a thin palladium film–aluminum electrode: analysis of some uranium mineral ores. Radiochimica Acta.

[CR33] Sander S (1999). Simultaneous adsorptive stripping voltammetric determination of molybdenum(VI), uranium(VI), vanadium(V), and antimony(III). Analytica Chimica Acta.

[CR34] Semião AJC, Rossiter HMA, Schäfer AI (2010). Impact of organic matter and speciation on the behavior of uranium in submerged ultrafiltration. Journal of Membrane Science.

[CR35] Shams E (2001). Determination of trace amount of bismuth(III) by adsorptive stripping voltammetry by Alizarine Red S. Electroanalysis.

[CR36] Shemirani F, Baghdadi M, Ramezani M, Jamali MR (2005). Determination of ultra trace amounts of bismuth in biological and water samples by electrothermal atomic absorption spectrometry (ET-AAS) after cloud point extraction. Analytica Chimica Acta.

[CR37] Stoltenberga M, Danschera G, Pamphlettb R, Christensena MM, Rungbya J (2000). Histochemical tracing of bismuth in testis from rats exposed intraperitoneally to bismuth subnitrate. Reproductive Toxicology.

[CR38] Sun M, Wu Q (2011). Determination of trace bismuth in human serum by cloud point extraction coupled flow injection inductively coupled plasma optical emission spectrometry. Journal of Hazardous Materials.

[CR39] Takata H, Aono T, Tagami K, Uchida S (2011). Determination of naturally occurring uranium concentrations in seawater, sediment, and marine organisms in Japanese estuarine areas. Journal of Radioanalytical and Nuclear Chemistry.

[CR40] Wang J (1985). Stripping analysis, principles, instrumentation and applications.

[CR41] Wang E, Su W, Yang Y (1984). Potentiometric stripping analysis with a thin-film gold electrode for determination of copper, bismuth, antimony, and lead. Analytical Chemistry.

[CR42] Wang J, Lu J, Wang J, Luo D, Tian B (1997). Simultaneous measurements of trace chromium and uranium using mixed ligand adsorptive stripping analysis. Analytica Chimica Acta.

[CR43] Wang J, Wang J, Tian B, Jiang M (1997). Adsorptive stripping measurements of chromium and uranium at iridium-based mercury electrodes. Analytical Chemistry.

[CR44] Zhang Y, Adeloju SB (2008). Flow injection–hydride generation atomic absorption spectrometric determination of selenium, arsenic and bismuth. Talanta.

